# Analysis on the muscle activity of antebrachial area at the iron swing of male high school golf players

**DOI:** 10.1186/1757-1146-7-S1-A112

**Published:** 2014-04-08

**Authors:** Oh Cheong Hwan, Hong Soo Young, Shin Eui Su, Bea Jae Hee

**Affiliations:** 1 Physical Education, Chungnam National Univ., Gung-dong, Yuseong-gu, Daejeon, 305-764, Korea

## Introduction

The purpose of this study is to provide systematic as well as scientific foundational data through 3D image analysis and electromyography (EMG) on male high school golf players’ #7 iron swing.

## Methods

The subjects selected for the experiment of this research were one player who had won in the male high school golf game and seven players whose golf career lasted for over five years. And their age (years) was 19.00±0.00, stature (m) was 1.73±3.29, body mass (Kg) was 79.00±13.25, and career (years) was 7.7±1.72. The equipment used for this research for photographing was nine infrared high-speed cameras (Motion Master 100, KOR), and the Kwon3d XP program was utilized for 3D motion analysis. And electromyography (EMG) (Tellemyo 2400 GT, USA) was used to analyze their right and left arms’ radial extensor of wrist and radial flexor of wrist muscle activity. Also, SPSS 21.0 was employed to conduct independent T-test.

## Results and discussion

Total time consumed for the male high school golf players’ #7 iron swing was 0.35±0.01 S. Advanced researches targeting professional golf players report that total time consumed at #7 iron swing is 0.33±0.01 S; thus, there is 0.02 S difference between amateur and professional golf players. This seems to be resulted from difference in the experimental environment, and the result is similar to that of advanced researches.

The displacement of the center of the body was found to be 0.02m in the pre- and post-variable X-axis. And averagely, it was 0.01m in the left and right variable Y-axis and was 0.08m in the horizontal and vertical variable Z-axis. Therefore, according to the result of comparing them with professional golf players in advanced researches, the pre- and post-variable X-axis indicated 0.03m difference, and the left and right variable Y-axis showed 0.01m difference averagely, and next, the horizontal and vertical variable Z-axis had no difference as average 0. This implies that professional golf players tend to hit the ball more efficiently than amateur golf players.

Kim Jae-sam (2009)’s research reports that at the #7 iron swing of professional golf players, the club head’s synthetic rate was found to be 25.73±0.33 m/s at the impact (E5). But in this research, it was calculated as 25.63±1.25 m/s at the impact (E5), so there was 0.1m/s or so difference. This means that professional golf players hit the ball slightly faster than amateur golf players.

Kim Chang-uk and Pak Jong-jin (2001)’s research compares five high school players with the club by analyzing the muscle activity of their arms at #7 iron golf swing. And according to the analysis on their left and right muscle groups, there was no statistically significant difference found; however, in this research, at the #7 iron golf swing of male high school golf players, the right arm’s radial extensor of wrist and radial flexor of wrist were higher than the left arm’s (p>.001).

## Conclusion

Therefore, they tend to use their right arm’s radial extensor of wrist and radial flexor of wrist more at the golf swing. This means that they use their right arm’s antebrachial area for hitting so that they can hit the ball more powerfully. If further analysis is done in consideration of each individual’s characteristics, it will help improve their performance in the game.
